# Predicting Outcome in dogs with Primary Immune‐Mediated Hemolytic Anemia: Results of a Multicenter Case Registry

**DOI:** 10.1111/jvim.13642

**Published:** 2015-10-16

**Authors:** R. Goggs, S.G. Dennis, A. Di Bella, K.R. Humm, G. McLauchlan, C. Mooney, A. Ridyard, S. Tappin, D. Walker, S. Warman, N.T. Whitley, D.C. Brodbelt, D.L. Chan

**Affiliations:** ^1^ School of Physiology and PharmacologyUniversity of BristolBristolUK; ^2^ Department of Clinical StudiesSchool of Veterinary MedicineUniversity of PennsylvaniaPhiladelphiaPA; ^3^ Vets Now Referrals KentBlue Bell HillUK; ^4^ Department of Clinical Sciences and ServicesRVCNorth MymmsUK; ^5^ University of GlasgowGlasgowUK; ^6^ University College DublinDublinIreland; ^7^ University of EdinburghEdinburghUK; ^8^ Dick White ReferralsSix Mile BottomUK; ^9^ Anderson Moores Veterinary SpecialistsWinchesterUK; ^10^ Companion Animal StudiesUniversity of BristolLangfordUK; ^11^ Davies Veterinary SpecialistsHigham GobionUK; ^12^ Production and Population HealthRoyal Veterinary CollegeNorth MymmsUK

**Keywords:** Canine hemolytic anemia objective score, Immune‐mediated hemolytic anemia, Survival, Thromboembolism

## Abstract

**Background:**

Outcome prediction in dogs with immune‐mediated hemolytic anemia (IMHA) is challenging and few prognostic indicators have been consistently identified.

**Objectives:**

An online case registry was initiated to: prospectively survey canine IMHA presentation and management in the British Isles; evaluate 2 previously reported illness severity scores, Canine Hemolytic Anemia Score (CHAOS) and *Toky*o and to identify independent prognostic markers.

**Animals:**

Data from 276 dogs with primary IMHA across 10 referral centers were collected between 2008 and 2012.

**Methods:**

Outcome prediction by previously reported illness‐severity scores was tested using univariate logistic regression. Independent predictors of death in hospital or by 30‐days after admission were identified using multivariable logistic regression.

**Results:**

Purebreds represented 89.1% dogs (n = 246). Immunosuppressive medications were administered to 88.4% dogs (n = 244), 76.1% (n = 210) received antithrombotics and 74.3% (n = 205) received packed red blood cells. Seventy‐four per cent of dogs (n = 205) were discharged from hospital and 67.7% (n = 187) were alive 30‐days after admission. Two dogs were lost to follow‐up at 30‐days. In univariate analyses CHAOS was associated with death in hospital and death within 30‐days. *Tokyo* score was not associated with either outcome measure. A model containing SIRS‐classification, ASA classification, ALT, bilirubin, urea and creatinine predicting outcome at discharge was accurate in 82% of cases. ASA classification, bilirubin, urea and creatinine were independently associated with death in hospital or by 30‐days.

**Conclusions and clinical importance:**

Markers of kidney function, bilirubin concentration and ASA classification are independently associated with outcome in dogs with IMHA. Validation of this score in an unrelated population is now warranted.

**Abbreviations**
ALP: alkaline phosphatase activity
ALT: alanine aminotransferase
ASA: American Society of Anesthesiologists
AUROC: area under the receiver‐operating curve
CHAOS: canine hemolytic anemia objective score
CI: confidence interval
DEA: dog erythrocyte antigen
hIVIG: human intravenous immunoglobulin
IMHA: immune‐mediated hemolytic anemia
MMF: mycophenolate mofetil
OR: odds ratio
ROC: receiver‐operating curve
SE: standard error
SIRS: systemic inflammatory response syndrome
UFH: unfractionated heparin

Immune‐mediated hemolytic anemia (IMHA) is among the most common autoimmune condition affecting dogs,^1^ and some aspects of its pathogenesis have been well characterized.^2^, ^3^ Despite such insights, the prognosis for dogs with IMHA remains guarded, with published case fatality rates for primary IMHA in dogs ranging from 26% to 60%.^4^, ^5^, ^6^ Previous studies have linked various clinicopathologic abnormalities with outcome in dogs with IMHA. Few prognostic indicators are consistent across multiple studies, however, perhaps because of differences between study populations or because of a lack of standardization. It has been suggested that validation and standardization of diagnostic criteria is urgently required for dogs with IMHA and that future interventional clinical trials would benefit from stratification by mortality risk.^7^ Mortality risk assessment for clinical trials is typically performed using illness severity scores.^8^ Accurate prognostication in a complex disease process like IMHA might require a multifaceted scoring system. Two such schemes, the canine hemolytic anemia objective score (CHAOS) and a score developed in Japan (*Tokyo*) have been proposed,^1^ ^9^ but neither has been independently evaluated to determine if they remain prognostic outside of the populations from which they were generated.

Alternatives to these disease‐specific illness severity scores that might be easier to estimate are the American Society of Anesthesiologists (ASA) health classification and the presence or absence of markers of a systemic inflammatory response syndrome (SIRS). The ASA classification is typically used to evaluate patient risk for anesthesia,^10^ but the classification is easy to apply and has been used as a marker of disease severity in other canine populations.^11^ The inflammatory response associated with IMHA in dogs is well‐recognized and can be evaluated through measurement of acute phase proteins,^12^ or cytokine concentrations.^13^ These measures are not widely available however, while a SIRS score based on readily obtained clinical data is a more universal means to identify dogs with systemic inflammation.^14^

Several studies from individual centers in the United Kingdom have been published recently, but each described relatively few cases and studied distinct aspects of the disease. Even with the benefit of these data, it is difficult to summarize the demographics, therapies and outcomes of the overall UK canine IMHA population presenting to referral centers.^15^, ^16^, ^17^

In this study we aimed to address these knowledge gaps by surveying case presentations, management strategies and outcomes of dogs with IMHA presenting to multiple referral centers in the British Isles. In addition, we aimed to test the association of illness‐severity markers ASA and SIRS status with outcome and test the predictive ability of 2 previously published IMHA‐specific illness severity scores. We also aimed to identify independent prognostic markers from our own dataset using a multivariate analysis approach and hypothesized that a multivariable scoring system would predict survival better than individual variables alone.

## Materials and Methods

### Sample Size

Based on previous publications we estimated case fatality at discharge at 17%,^16^ and that 20% dogs would have previously identified risk factors.^6^, ^18^, ^19^ We aimed to detect a 2‐fold increase in case fatality risk where such factors were present and therefore planned to enroll 335 dogs.^2^

### Case Recruitment

Collaborators were recruited by publication of a letter inviting participation,^20^ and through direct contact with referral centers. Data sharing was agreed in writing. Cases admitted from January 1, 2008 to December 31, 2009 were included retrospectively. Dogs admitted between January 1, 2010 and December 31, 2012 were enrolled prospectively. Dogs with primary, idiopathic IMHA admitted to participating institutions within the study period were eligible for inclusion. To maximize recruitment, the following previously published diagnostic criteria were used:^13^, ^16^ anemia (PCV<37%) AND at least one of the following: positive in‐saline agglutination test, OR a positive Coombs' test, OR moderate‐marked spherocytosis identified by a board‐certified clinical pathologist. Dogs were excluded if evidence of a predisposing disease process was present.^21^ All dogs underwent diagnostic evaluation according to their individual case histories as judged appropriate by their attending primary clinicians. These diagnostic evaluations (summarized in Table S1) were not standardized, but aimed to identify potential underlying causes and typically included CBC, serum biochemistry, thoracic, and abdominal imaging, PCR testing for tick‐borne infections by *Babesia*,* Ehrlichia*, and *Mycoplasma* species and urine culture. Attending clinicians determined case management.

### Data Acquisition and Handling

Study data (Data S1) were collected using secure, web‐based software that enabled automated data export.^3^ Historical, demographic, at‐admission clinicopathologic, treatment, and outcome data were recorded via a custom survey, accessible from January 1, 2010 to February 1, 2013, agreed in advance by all participating centers (Data S2). The survey used dropdown menus, limited‐response questions and constrained textboxes to minimize errors. Free‐text boxes enabled addition of contextual comments to aid interpretation. Raw data were regularly inspected and where necessary, centers were contacted to correct erroneous or incomplete entries. ASA status was assigned as follows: Grade 1, Normal; Grade 2, Mild systemic disease; Grade 3, Severe systemic disease; Grade 4, Life‐threatening systemic disease; Grade 5, Moribund patient, not expected to survive.^22^ Illness severity scores CHAOS,^1^ and *Tokyo,* 9 were calculated as previously reported (Table 1). Systemic inflammatory response syndrome (SIRS) was diagnosed using published criteria: Temperature ≤100.0°F or ≥103.5°F; heart rate >160 bpm, RR >40 bpm; leukocyte count ≤4,000/μL or ≥12,000/μL or ≥10% band neutrophils.^23^ Anisocytosis and polychromasia were graded as mild, moderate, or severe as previously described. Similarly, spherocytes were quantified in the monolayer using a 1+ to 3+ scale, where 1+ equals 5–10 spherocytes per 100× oil field (2–4% of the RBCs); 2+ equals 11–50 (4–20%); and 3+ equals 51–150 spherocytes per field (20–60%).^21^ Where discrepancies between automated and manual platelet counts occurred, manual counts were used for calculations. Saline agglutination tests were performed using a drop of EDTA‐anticoagulated blood mixed with a drop of saline on a microscope slide and examined against a white background over a period of 1–2 minutes for gross agglutination, followed by microscopic evaluation for differentiation from rouleaux.

**Table 1:** Calculation of CHAOS and *Tokyo* illness severity scores

Canine hemolytic anemic objective score (CHAOS)	
Age (year)	If ≥7 score 2, otherwise score 0
Temperature (°F)	If ≥102.0 score 1, otherwise score 0
Agglutination	If present score 1, otherwise score 0
Albumin (g/dL)	If <3.0 score 1, otherwise score 0
Bilirubin (mg/dL)	If ≥5.0 score 2, otherwise score 0
Total	Maximum score 7

*Tokyo* score	
Sex	Male score 1, Female score 0
Season	Apr‐Sept score 1, Oct‐Mar score 0
Packed cell volume (%)	If <20 score 1, otherwise score 0
Platelet count (× 10^3^/μL)	If <200 score 1, otherwise score 0
Total protein (g/dL)	If <6.0 score 1, otherwise score 0
Total	Maximum score 5

### Data Analysis

Exported data were collated and analyzed using proprietary software.^4^^,^ 5 ^,^ 6 Data were assessed for normality prior to test selection. Although some variables were parametric, most were not, thus variables are reported as median (interquartile range). To validate use of the whole dataset for outcome analyses, retrospective, and prospective data were compared using nonparametric tests. To correct for multiple (*m*) comparisons while minimizing the risk of dismissing significant differences, the *P*‐value was adjusted: *P*_*adjusted*_= [0.05 x (*m*+1)] / (2*m*).^24^ CHAOS, *Tokyo*, case, treatment, and center variables were tested for association with death during hospitalization and death by 30 days by univariate logistic regression. The effect of center was assessed using multiple dichotomous variables with a reference group.

Multivariable logistic regression using case variables was then undertaken to generate prognostic models. Center and treatment were excluded from the prognostic models because they were not generalizable to other populations, and were potentially subject to bias from financial constraint and clinician preference respectively. Previously, reported illness‐severity scores (CHAOS and *Tokyo*) were not included in the multivariable analyses. Candidate predictor variables were chosen as follows: associated with outcome in the univariate regression at *P* < .1; no evidence of collinearity (correlation coefficient <0.9); an event:variable ratio >5.^25^ For a complete case analysis to be performed in the prognostic models, only variables with <5% missing data were included.^26^ Alanine aminotransferase (ALT), bilirubin, urea, and creatinine values were indexed against (divided by) each center's upper reference interval to account for variations in reference ranges. All variables were simultaneously entered into the model to maximize the predictive ability of prognostic models. Model accuracy was determined using 2 × 2 classification tables. Model discrimination was determined by calculating area under the receiver‐operating characteristic curve (AUROC). Model calibration was assessed by Hosmer–Lemeshow goodness‐of‐fit (model rejected if *P* < .05) and visual inspection of the contingency table. Model utility was assessed using Nagelkerke's *R* ^2^.

## Results

### Retrospective and Prospective Case Comparisons

Although our aim was to enroll 335 cases, the rate of case recruitment was slower than anticipated. To minimize time‐dependent changes in case management, the registry was closed early, limiting the study period to 5 years. Data from 276 cases (215 prospective, 61 retrospective) were collected. Only 3 variables differed significantly between retrospective and prospective populations (Table 2), which were therefore considered sufficiently comparable for subsequent combined analyses.

**Table 2:** Descriptive statistics of clinicopathologic data for the retrospective, prospective and combined populations. Variables significantly different between the prospective and retrospective populations (*P* < .05 after adjustment for multiple comparisons) are indicated in bold typeface

Variable	Retrospective		Prospective		Comparison	Combined	
	Median (IQR)	n	Median (IQR)	n	*P*‐value	Median (IQR)	n
Age	7 (5)	61	6 (4)	214	.25	6 (4.1)	275
ASA	3 (1)	51	3 (1)	215	.73	3 (1)	266
Heart rate	128 (34)	61	130 (39)	208	.43	130 (33)	269
Resp. rate	36 (24)	55	36 (16)	182	.06	36 (19)	237
Temperature	101.3 (1.5)	60	101.7 (1.6)	205	.07	101.7 (1.6)	265
Albumin	2.8 (0.7)	59	2.9 (0.6)	209	.86	2.9 (0.6)	268
Globulin	3.4 (1.2)	59	3.3 (1.0)	209	.89	3.3 (1.1)	268
ALT	65 (195)	59	44 (85)	209	**.03**	45 (98)	268
ALP	198 (274)	59	184 (229)	210	.08	189 (246)	269
Bilirubin	27.4 (81.6)	58	16.0 (53.5)	207	.09	17.1 (59)	265
Urea	8.1 (6.3)	59	7.5 (5.1)	210	.63	7.6 (5.3)	269
Creatinine	65 (24)	59	66.5 (28)	206	.44	66 (27)	265
PCV	14.5 (8.0)	61	14.0 (7.0)	215	.35	14.0 (7.2)	276
Leukocytes	26.5 (20.9)	61	21.6 (21)	213	.17	22.5 (21.4)	274
Neutrophils	19.3 (17.1)	61	16.0 (17.2)	214	.15	16.9 (17.2)	275
Monocytes	2.4 (3.7)	61	1.8 (3.0)	214	.08	2.0 (3.0)	275
Platelet count	200 (72)	57	186 (173)	213	.86	187 (147)	270
Retics	160.0 (234)	43	129.5 (224)	137	.15	137 (226)	180
CHAOS	3 (2)	57	3 (2)	215	.05	3 (2)	272
*Tokyo*	3 (1)	57	3 (1)	215	.31	3 (1)	272
Polychromasia	2 (1)	57	2 (2)	199	.09	2 (1)	256
Anisocytosis	3 (1)	38	2 (1)	204	**<.01**	2 (1)	242
Spherocytosis	2 (2)	58	2 (2)	196	.57	2 (2)	254

Breed	Breed variation *P* = .57
Sex	Sex variation *P* > .99
Drug (1st)	1st immunosuppressive drug variation *P* = .96
Drug (2nd)	2nd immunosuppressive drug variation *P* = .94
Antithrombotics	Antithrombotic drug variation *P* = .84

	+/−	n	+/−	n	*P*‐value	+/−	n
Agglutination	50/11	61	170/45	215	.72	220/56	276
Icterus	31/27	58	75/135	210	**.02**	106/162	268
Pigmenturia	15/16	31	57/103	160	.23	72/119	191
Travel	0/61	61	14/201	215	.05	14/262	276
Medication	19/42	61	98/115	213	.04	117/157	274
Vaccination	2/59	61	11/204	215	.74	13/263	276
Discharged	46/15	61	159/56	215	.87	205/71	276
Alive at 30d	43/17	60	143/71	214	.53	186/88	274

ASA, American Society of Anesthesiologists physical status classification; ALT, alanine transaminase activity; ALP, alkaline phosphatase activity; Retics, absolute reticulocyte count. *P* < .03 were considered significant at the *P* < .05 level after adjustment for multiple comparisons.

### Study Population Characteristics

All centers contributed cases, median 18 (29) per center. There were 246 (89.1%) purebred dogs and 30 (10.9%) cross‐breeds. Two breeds were particularly prevalent: springer spaniels (n = 46, 16.7%) and cocker spaniels (n = 46, 16.7%). Spayed females represented 47.4% (n = 131), entire females 13.8% (n = 38), castrated males 27.2% (n = 75) and entire males 11.6% (n = 32). Fourteen dogs (5.1%) had a foreign‐travel history. Thirteen dogs (4.7%) were vaccinated within 30 days of presentation. Many cases (n = 121, 43.8%) received medication in the month before presentation, typically for clinical signs attributable to IMHA: antibiotics (n = 63, 22.8%), glucocorticoids (n = 46, 16.7%), nonsteroidal anti‐inflammatory drugs (n = 31, 11.2%), and immunosuppressives (n = 13, 4.7%). Therapies for chronic disorders including epilepsy, diabetes mellitus, or hypothyroidism were administered to 31 dogs (11.2%).

### Clinicopathologic Data

Two hundred twenty dogs were in‐saline agglutination positive (79.7%), 85/113 dogs (75.2%) with a Coombs test result were positive, 183/254 (72.0%) dogs had moderate‐marked spherocytosis. Blood typing for DEA 1 was attempted in 212 dogs (76.8%) and established in 180 (65.2%): 95 (34.4%) were positive and 85 (30.8%) negative. The median ASA classification was 3 (2–3), the median CHAOS was 3 (2–4) and the median *Tokyo* score was 3 (2–3); 58.3% fulfilled 2/4 SIRS criteria (n = 161), 19.9% fulfilled 3/4 SIRS criteria (n = 55).

### Treatment

Most dogs (n = 244, 88.4%) received primary immunosuppressives, 101 dogs (36.6%) received 2 first‐line drugs and 9 (3.3%) received 3 (Table 3). Second‐line immunosuppressives were used in 156 dogs (56.5%), 46 dogs (16.6%) received 2 additional drugs, and 7 (2.5%) received 3. Second‐line drugs were instituted on median day 3 (2–5) after admission. Antithrombotics were administered to 210 dogs (76.0%) including: aspirin (n = 182, 65.9%), low molecular‐weight heparin (n = 31, 11.1%), clopidogrel (n = 23, 8.3%) and unfractionated heparin (n = 10, 3.6%). Multiple antithrombotics were administered to 32 dogs (11.6%). Packed red blood cells were administered to 205 dogs (74.3%) (125 received 1 unit, 53 received 2 and 26 received ≥3). Fresh whole blood was administered to 21 dogs (7.6%) (17 received 1 unit and 4 received 2). Hemoglobin‐based oxygen carrying solution was administered to 32 dogs (11.6%) (18 received 1 unit, 10 received 2, and 4 received 3). Only 5 dogs received fresh frozen plasma. More than 1 product type was administered to 35 dogs (12.7%).

**Table 3:** Summary of immunosuppressive and antithrombotic therapies administered to the study population

First‐line drug	n	% total pop.	Median (min–max) dose	Mode frequency
Dexamethasone	121	43.8	0.3 mg/kg (0.15–2)	Q24 h
Prednisolone	121	43.7	1.3 mg/kg (0.5–2.2)	Q12 h
Azathioprine	78	28.2	2 mg/kg (1.23–2.5)	Q24 h
Cyclosporine	30	10.9	5 mg/kg (3–7)	Q24 h
hIVIG	5	1.8	0.5 g/kg (0.3–0.6)	Q24 h
MMF	4	1.4	15 mg/kg (13–15)	Q12 h

Second‐line drug	n	% total pop.	Median (min–max) dose	Mode frequency
Prednisolone	78	28.2	1 mg/kg (0.65–2)	Q12 h
Azathioprine	39	14.1	2 mg/kg (1–4)	Q24 h
Cyclosporine	29	10.5	3.3 mg/kg (2–7)	Q12 h
MMF	10	3.6	10 mg/kg (5–20)	Q12 h
hIVIG	6	2.2	0.5 g/kg (0.2–0.5)	Q24 h
Cytarabine	3	1.1	50 mg/m^2^ (50–100)	Q12 h
Dexamethasone	1	0.4	0.3 mg/kg	Q24 h
Cyclophosphamide	1	0.4	5 mg/kg	Q12 h

Antithrombotic	n	% total pop.	Median (min–max) dose	Mode frequency
Aspirin	182	65.9	0.5 mg/kg (0.25–5)	Q24 h
Dalteparin	31	11.2	150 IU/kg (100–250)	Q8 h
Clopidogrel	23	8.3	2 mg/kg (0.25–4.5)	Q24 h
UFH	10	3.6	200 IU/kg (75–200)	Q8 h

hIVIG, human immunoglobulin g; MMF, mycophenolate mofetil; UFH, unfractionated heparin.

### Survival

Two hundred and five (74.3%) dogs were discharged from the hospital, equivalent to 25.7% mortality at discharge. Of the 71 nonsurvivors, 56 (20.3%) were euthanized and 15 (5.4%) died. Sixteen dogs (5.8%) were discharged but subsequently were euthanized or died and 2 dogs were lost to follow‐up, such that 186 (67.4%) dogs were alive at 30 days after admission, equivalent to 30‐day mortality of 32.6%. Twelve dogs underwent necropsy and no underlying diseases were identified.

### Predictive Value of CHAOS and Tokyo

In univariate analyses CHAOS, when dichotomized as <3 or ≥3, was associated with death in hospital and death within 30 days of admission. *Tokyo* score, when dichotomized as <3 or ≥3, was not associated with any of the 3 outcome measures (Table 4). ROC curve data for CHAOS, *Tokyo*, and the prognostic score from the multivariable models are reported in Table 5. The AUROC point estimate and 95% confidence intervals for CHAOS scores were higher than those for *Tokyo* scores. The 95% confidence intervals for *Tokyo* scores all included 0.5, suggesting it was little better than chance at predicting outcome in this population.

**Table 4:** Results of univariate analyses for association with death at discharge and death within 30 days of admission. Variables significantly associated with the outcome variable (*P* < .1) are indicated in bold typeface

Variable	Dead at discharge				Dead at 30‐days			
	*P* value	OR	95% CI of OR		*P* value	OR	95% CI of OR	
			Lower	Upper			Lower	Upper
CHAOS score ≥3	**<.01**	**4.162**	**2.178**	**7.951**	**<.01**	**3.560**	**1.998**	**6.341**
*Tokyo* score	.99	1.004	0.577	1.749	.22	1.391	0.818	2.365
SIRS ≥3/4 criteria	**.03**	**2.040**	**1.091**	**3.816**	**.04**	**1.917**	**1.044**	**3.521**
ASA ≥3	**<.01**	**3.205**	**1.541**	**6.665**	**<.01**	**2.663**	**1.411**	**5.027**
Respiratory rate	**.20**	**1.010**	**0.995**	**1.026**	.35	1.007	0.992	1.023
Pigmenturia	**<.01**	**2.536**	**1.330**	**4.834**	**.01**	**2.184**	**1.186**	**4.024**
Autoagglutination	.97	0.985	0.465	2.089	.98	0.991	0.484	2.029
ALT (indexed)	**.05**	**1.040**	**1.001**	**1.081**	**.06**	**1.039**	**0.999**	**1.081**
Bilirubin (indexed)	**<.01**	**1.013**	**1.005**	**1.020**	**<.01**	**1.011**	**1.004**	**1.018**
Urea (indexed)	**<.01**	**3.135**	**1.955**	**5.029**	**<.01**	**2.587**	**1.666**	**4.018**
Creatinine (indexed)	**.06**	**2.356**	**0.956**	**5.804**	**<.01**	**9.892**	**2.677**	**36.548**

CHAOS, canine hemolytic anemia objective score; SIRS, systemic inflammatory response syndrome; Abbreviations, ASA, American Society of Anesthesiologists physical status classification; ALT, alanine transaminase activity; OR, odds ratio.

**Table 5:** Comparison of the abilities of the Study model, CHAOS and *Tokyo* scores to predict death at discharge and day 30. Receiver operating characteristic curve results for the Study model, CHAOS and *Tokyo* scores

Death at discharge					
Score	AUROC	SE	Asymptotic significance	Asymptotic 95% CI	
				Lower	Upper
Study model	0.775	0.035	<0.001	0.706	0.844
CHAOS	0.688	0.036	<0.001	0.618	0.758
*Tokyo*	0.519	0.040	0.641	0.440	0.598

Death at day 30					
Score	AUROC	SE	Asymptotic significance	Asymptotic 95% CI	
				Lower	Upper
Study model	0.729	0.036	<0.001	0.657	0.800
CHAOS	0.688	0.034	<0.001	0.622	0.755
*Tokyo*	0.542	0.037	0.261	0.470	0.615

AUROC, area under the receiver operating characteristic curve; SE, standard error; 95% CI, confidence intervals for odds ratio.

### Outcome Modeling

In univariate analyses, 8 candidate variables were associated with both survival at discharge and survival at 30 days (Table 4). There was no association between center and death at discharge or at 30 days. Two variables were excluded for having >5% missing cases. Center and the CHAOS and *Tokyo* scores were deliberately excluded from multivariable modeling. For survival prediction multivariable analyses, 6 variables were entered into the final model: SIRS, ASA classification, ALT, bilirubin, urea, and creatinine. For prediction of outcome at discharge, this model was accurate in 82% of cases. In multivariate analysis, 3 variables were independently predictive of death in hospital: ASA classification, bilirubin, and urea. Three variables were independently predictive of death by day 30: ASA classification, bilirubin, and creatinine (Table 6).

**Table 6:** Summary of multivariable logistic regression models. Variables significantly associated with the outcome variable (*P* < .05) are indicated in bold typeface

Summary of independent predictive variables for death at discharge						
Variable	Coefficient (B)	SE	*P* value	Odds ratio (OR)	95% CI for OR	
					Lower	Upper
SIRS ≥3/4 criteria	0.233	0.410	.57	1.263	0.565	2.823
**ASA ≥3**	**0.997**	**0.475**	**.04**	**2.709**	**1.068**	**6.870**
ALT (indexed)	0.028	0.019	.15	1.028	0.990	1.067
**Bilirubin (indexed)**	**0.010**	**0.004**	**<.01**	**1.010**	**1.003**	**1.017**
**Urea (indexed)**	**1.114**	**0.331**	**<.01**	**3.046**	**1.592**	**5.830**
Creatinine (indexed)	−0.639	0.588	.28	0.528	0.167	1.670
Constant	3.333	0.507				

AUROC	0.775 (95% CI 0.706–0.844)
Hosmer–Lemeshow *P* value	.31
Nagelkerke's R^2^	0.304

Summary of independent predictive variables for death at 30‐days						
Variable	Coefficient (B)	SE	*P* value	Odds ratio (OR)	95% CI for OR	
					Lower	Upper
SIRS ≥3/4 criteria	0.201	0.384	.60	1.222	0.576	2.593
**ASA ≥3**	**0.776**	**0.386**	**.04**	**2.173**	**1.020**	**4.629**
ALT (indexed)	0.025	0.018	.17	1.026	0.989	1.064
**Bilirubin (indexed)**	**0.009**	**0.003**	**.01**	**1.009**	**1.002**	**1.016**
Urea (indexed)	0.440	0.274	.11	1.552	0.907	2.655
**Creatinine (indexed)**	**2.197**	**0.902**	**.02**	**8.996**	**1.537**	**52.672**
Constant	−3.449	0.579				

AUROC	0.729 (95% CI 0.657–0.800)
Hosmer–Lemeshow *P* value	.89
Nagelkerke's *R* ^2^	0.261

SE, standard error; 95% CI, confidence intervals for odds ratio; AUROC, area under the receiver operating characteristic probability curve; OR, odds ratio.

## Discussion

This multicenter study provides an overview of case characteristics, management, and outcome for 276 dogs with primary IMHA treated at referral centers in the British Isles between 2008 and 2012. Despite intensive management with immunosuppressives, blood products, and antithrombotics, the 30‐day mortality rate was 32.6%. This figure is comparable to previous studies,^4^, ^7^, ^27^, ^28^ perhaps suggesting our ability to treat IMHA has not improved in recent years.

Illness‐severity scores might help identify dogs that might benefit from treatment intensification. This study evaluated the association of 2 IMHA specific illness‐severity scores with outcome in our population. Of these, CHAOS ≥3 was associated with increased odds of death and in particular, a high CHAOS was associated with a risk of death during hospitalization. *Tokyo* score was not useful for outcome prediction in our study. We also found that ASA classification ≥3 was also associated with death, suggesting the subjective assessment of experienced clinicians can be a reasonable gauge of illness severity in IMHA. As can be seen from the AUROC values (Table 5), the final multivariate model allows outcome to be predicted more accurately than previously reported scoring systems. This is not unexpected, since a model generated from our data should describe our population better than those derived from other populations, and independent evaluation of our model in an unrelated population should be undertaken to ensure its validity. It is noteworthy however that of the 2 previously developed scores, the AUROC point estimate and 95% confidence intervals for CHAOS were good, while the 95% confidence intervals for *Tokyo* included 0.5, suggesting it was little better than chance at predicting outcome in our population.

We used logistic regression analysis to assess the association of individual clinical and clinicopathologic variables with outcome. Individual variables with significant associations with outcome were combined into multivariable models and the accuracy of these models evaluated using 2 × 2 classification tables that identify how many dogs were correctly classified as dead or alive. The AUROC values were higher for the final multivariable model than for any of the individual variables alone (data not shown). The final 6‐variable model for prediction of outcome at discharge was accurate in 82% cases. Although this suggests the model was highly accurate, it should be noted that assuming every dog was discharged alive would have been correct in 74% of cases. The *R* ^2^ values suggest that the 6 variables (SIRS, ASA classification, ALT, bilirubin, urea, and creatinine) included in our death in hospital model represent only a minority of the factors determining outcome. In linear regression, a model containing all the variables needed to explain outcome has *R* ^2^ = 1. While *R* ^2^ values in logistic regression are pseudo‐*R* ^2^, their interpretation is similar. For example, our 6‐variable model predicting death during hospitalization had *R* ^2^ = 0.304. This indicates that most of the factors that influence the likelihood of death during hospitalization were not included in our model. These unquantified variables might be unidentified or unmeasured case factors, the effects of treatment and complications including thrombosis.

Seventy‐two dogs died before discharge, while a further 16 did not survive to 30 days. Three variables were predictive of outcome at both times, suggesting some consistency between the causes of death during hospitalization and at 30 days. There was 1 difference in the prediction models between outcomes at discharge versus 30 days: urea was independently predictive of outcome at discharge but not at 30 days, while creatinine was not independently predictive of outcome at discharge but was at 30 days. The cause of these differences is unclear. The association between creatinine concentration and outcome at 30 days might suggest that end‐organ dysfunction associated with hypoxemia, nephrotoxicity from drug administration, or hemoglobinemia affects medium‐term outcome. Acute kidney injury is associated with nonsurvival in critically‐ill dogs,^29^ and even small deteriorations in kidney function can affect outcome.^30^

Our intention was to test our hypotheses by enrolling 335 dogs; however, we were only successful in recruiting 276 dogs within a 5‐year period. Although this might have reduced our ability to identify reliable prognostic markers, post hoc power calculations suggested we were powered to detect a 2.02‐fold change in case fatality, which was almost exactly our goal. This study combined retrospective and prospectively collected data into 1 dataset, to maximize the data available for analysis, while minimizing the time taken for data collection. We compared demographics, clinicopathologic data, and treatment data for these populations prior to combining them. Three statistically significant differences were identified between these 2 populations (ALT activity, icterus frequency, and anisocytosis severity), but the clinical relevance of these differences is debatable. The absence of a difference in outcomes between the retrospective and the prospective groups supports this assertion. We cannot exclude the possibility that other differences might have existed that would affect the validity of our approach, but all data from the 2 parts of the study were collected from the same group of centers, which should improve the homogeneity of the data. Overall, we feel that the 2 populations were not clinically different sufficient that this would affect the results of our evaluation of associations using data from the combined population.

Our analyses attempted to account for differences in the reference intervals between centers. After initial screening, it was determined that some variables should be indexed to the institution's reference intervals. Since this was not performed for all variables, it is possible we might have overlooked some significant associations between nonindexed variables and outcome. However all of the variables in the final models were indexed, which maximizes their generalizability. We also considered that center might have had an effect on outcome either through distinct case demographics or institutional differences in case management or treatment availability. To address this, we evaluated the association of center with outcomes in univariate analyses. We found no significant effect of center on outcome in these analyses. Center was deliberately excluded from the multivariable analyses to maximize generalizability, but regardless center would not have been included in our multivariable models on the basis of a lack of association in the univariate analyses. This argues that if distinct treatment strategies employed by different institutions significantly influenced outcome then an effect of center on outcome might have been found, but was not. The effects of treatment on outcome were not evaluated by this study directly, but warrant investigation in prospective interventional trials.

Although tailored diagnostic evaluation was performed in all of the cases to identify an underlying mechanism for their IMHA, this was not exhaustive and thus we cannot exclude the possibility that some of these dogs had an unidentified primary cause. For instance, many dogs underwent PCR screening for vector‐borne pathogens, but few underwent more complete screening as has been recently recommended.^31^ The potential effect of this is difficult to quantitate, since unidentified primary causes of IMHA might be expected to worsen the prognosis by perpetuating the generation of autoantibodies. Other limitations inherent in this study are biases induced by financial limitations and euthanasia. Assessing dogs managed only at referral centers might have reduced these effects by reducing the number of dogs euthanized for financial limitations. Most deaths in this study were due to euthanasia, however, with the inherent potential for confounding by euthanasia for reasons other than illness‐severity or lack of response to treatment that is difficult to codify or exclude.

## Conclusion

This large multicenter cohort study provides insight into the current management and outcome of dogs with IMHA treated in the British Isles. Two previously published illness‐severity scores (CHAOS and *Tokyo*) were prospectively evaluated for their ability to predict outcome in a population separate from that used to generate the score. Of these two, only CHAOS was predictive of outcome in our population. Using our large dataset, we identified that markers of kidney function, bilirubin concentration, and ASA classification are independently associated with outcome in dogs with IMHA; a multivariate model combining illness severity scores and clinicopathologic data correctly predicted outcome at discharge in 82% cases. The ability of the factors identified here to predict outcome can now be evaluated in other populations, ideally before use as a means to stratify dogs for prospective interventional trials.

## Supporting information


**Table S1.** Summary of diagnostic testing undertaken to exclude primary causes of IMHA in the study population.

**Data S1.** Final database containing demographic, clinical, clinicopathologic, treatment and outcome data.

**Data S2.** A printable version of the custom online survey form used to facilitate data entry.

## Footnotes

1 Whelan MF, Rozanski EA, O'Toole TE, et al. Use of the canine hemolytic anemia objective score (CHAOS) to predict survival in dogs with immune mediated hemolytic anemia. J Vet Intern Med 2006; 20:714–715 [Abstract]

2
http://wwwn.cdc.gov/epiinfo/html/ei6_downloads.htm

3
https://www.surveymonkey.com

4 Excel 2010, Microsoft

5 SPSS 21, IBM

6 Prism 5.0, GraphPad
