# Supplementary material for: Predicting Outcome in dogs with Primary Immune‐Mediated Hemolytic Anemia: Results of a Multicenter Case Registry
Source: J Vet Intern Med. 2015 Oct 16;29(6):1603–10. doi: 10.1111/jvim.13642 (PMC4864895; doi:10.1111/jvim.13642)
Supplement: Supplementary file 1 — Table S1. Summary of diagnostic testing undertaken to exclude primary causes of IMHA in the study population. [file JVIM-29-1603-s001.pdf]

Table S1: Summary of diagnostic testing undertaken to exclude primary causes of IMHA in the study population. Abbreviations: PCR: polymerase chain reaction; MRI: magnetic resonance imaging; CSF: cerebrospinal fluid; FNA: fine-needle aspirate; PFK: phosphofructokinase; ACTH: adrenocorticotrophic hormone.

Diagnostic test	n	% population
Abdominal ultrasound	269	97.1
Thoracic radiography	258	93.1
Tick-borne disease PCR testing	137	49.5
Urine culture	131	47.3
Abdominal radiography	110	39.7
Splenic fine needle aspirates	25	9.0
Hepatic fine needle aspirates	19	6.9
Lymph node aspirates	19	6.9
Canine pancreatic lipase immunoreactivity	12	4.3
Bone marrow aspirate and core biopsy	11	4.0
Additional infectious disease testing	8	2.9
Fecal parasitology examination or culture	8	2.9
Anti-nuclear antibody testing	7	2.5
Computed tomography (any)	7	2.5
Echocardiography	7	2.5
Brain MRI and CSF sampling	4	1.4
Abdominocentesis and fluid analysis	3	1.1
FNA of other mass lesions	3	1.1
Serum protein electrophoresis	3	1.1
PFK DNA testing	2	0.7
ACTH-stimulation test	1	0.4
Arterial blood gas analysis	1	0.4
Blood cultures	1	0.4
Basal cortisol	1	0.4
Laparotomy	1	0.4
Total iron binding capacity	1	0.4
Thoracolumbar spine radiographs	1	0.4
Urinalysis without culture	1	0.4
Urine protein:creatinine ratio	1	0.4
Serum zinc and lead levels	1	0.4
