# Supplementary material for: Predicting Outcome in dogs with Primary Immune‐Mediated Hemolytic Anemia: Results of a Multicenter Case Registry
Source: J Vet Intern Med. 2015 Oct 16;29(6):1603–10. doi: 10.1111/jvim.13642 (PMC4864895; doi:10.1111/jvim.13642)
Supplement: Supplementary file 3 — Data S2. A printable version of the custom online survey form used to facilitate data entry. [file JVIM-29-1603-s003.pdf]

1. Inclusion / Exclusion Criteria

Welcome to the Canine IMHA Case Registry

Just as a reminder, here are the inclusion/exclusion criteria:

* * * * * *

Patient inclusion criteria: Any dog with a clinicopathologic diagnosis of primary IMHA based on:

Anaemia (PCV or HCT <37%) WITH

A positive in-saline agglutination test OR

A positive Coombs' test OR

Moderate to marked spherocytosis identified by a board certified clinical pathologist

* * * * * *

Specific exclusions: Evidence of an underlying disease process such as neoplasia predisposing to IMHA

2. Organisation

1. Please select your institution

2. Please enter your unique patient identifying number (case number)

3. What date was this patient admitted to your hospital? (DD/MM/YYYY)

3. Signalment

4. Patient age in years at initial hospital admission

5. Breed:

Please select from the list below

Other (please specify)

6. Sex

- Male entire
- Female entire
- Male castrated
- Female spayed

4. Potential IMHA triggers

7. Does this patient have a history of foreign travel?

- Yes
- No

If yes, please specify to where and when

8. Did this patient receive any medication in the 30 days prior to hospital admission?

- Yes
- No

If yes, please specify which drug(s) were administered and when

9. Was this patient vaccinated within 30 days of hospital admission?

- Yes
- No

If yes, please specify which vaccine was administered and when

5. Physical examination

10. Which ASA grade best describes the patient?

- ASA I (Normal, healthy)
- ASA II (Mild systemic disease)
- ASA III (Severe systemic disease)
- ASA IV (Life-threatening disease)
- ASA V (Close to death)

11. Initial physical examination findings

Pulse rate

Respiratory rate

Rectal temperature

Icterus (Y/N)

Pigmenturia (Y/N/Unknown)

6. Diagnostic confirmation

12. Which of these diagnostic tests were performed to rule out underlying causes?

- Thoracic radiography
- Hepatic biopsy (any type)
- Urine culture
- Abdominal radiography
- Splenic biopsy (any type)
- Tick borne disease screen
- Abdominal ultrasound
- LN biopsy (any type)
- ANA titres
- Other (please specify)

7. Clinicopathologic data

13. Serum biochemistry data:

Please record at admission (or first) values

Albumin (g/l)

Globulin (g/l)

ALT activity (U/l)

ALP activity (U/l)

Total bilirubin (umol/l)

Urea (mmol/l)

Creatinine (umol/l)

14. Complete blood count data:

Please enter at admission (or first) values

PCV (%) or Hct (%)

Total leukocyte count (x10e9/l)

Neutrophil count (x10e9/l)

Band neutrophil count (x10e9/l)

Monocyte count (x10e9/l)

Automated platelet count (x10e9/l)

Manual platelet count (if different)

Absolute reticulocyte count (cells/ul)

8. Clinicopathologic data

15. Degree of polychromasia

- Mild (1+)
- Marked (3+)
- None
- Moderate (2+)
- Rare
- Not assessed

16. Degree of anisocytosis

- Mild (1+)
- Marked (3+)
- None
- Moderate (2+)
- Rare
- Not assessed

17. Degree of spherocytosis

- Mild (1+)
- Marked (3+)
- None
- Moderate (2+)
- Rare
- Not assessed

18. Confirmed in-saline agglutination

- Present
- Absent
- Not performed

19. Coombs' test

- Positive
- Negative
- Not performed

Please enter any relevant details about the Coombs' test (e.g. IgG/IgM or 4oC/37oC or monoclonal/polyclonal)

20. Blood type

- DEA 1.1 Positive
- DEA 1.1 Negative
- Not performed
- Unable t

9. Coagulation parameters

21. Coagulation parameters and inflammatory markers: Please record at admission (or first) values

PT (s)

aPTT (s)

Fibrinogen (umol/l)

Antithrombin activity (%)

TAT complexes (ug/l)

CRP (mg/l)

22. D-Dimers: Please select the patient's at admission (or first) value from the list below

- <250 ng/ml
- 250-500 ng/ml
- 500-1000 ng/ml
- 1000-2000 ng/ml
- >2000 ng/ml

23. TEG parameters: Please record at admission (or first) values

R time (min)

K time (min)

alpha angle (o)

MA value (mm)

10. Treatment

24. First-line immunosuppressive drugs:
Please indicate the drugs and the dosages of all drugs used as first-line therapy in this patient

Azathioprine	
Ciclosporine	
Cyclophosphamide	
Danazol	
Dexamethasone	
hIVIG	
Leflunomide	
Mycophenolate mofetil	
Prednisolone	
Other (please specify)	

25. Second-line immunosuppressive drugs:
Please record drug dosages and day of initiation of all drugs used as second-line therapy in this patient

Azathioprine	
Ciclosporine	
Cylophosphamide	
Danazol	
Dexamethasone	
hIVIG	
Leflunomide	
Mycophenolate mofetil	
Prednisolone	
Other (please specify)	

11.

26. Blood products (Number of units administered)

	0	1	2	3	4	5	>5
Packed red blood cells							
Fresh whole blood							
Fresh frozen plasma							
HBOC (Oxyglobin)							

27. Thromboprophylactic drugs:

Please record which (if any) of these thromboprophylactic drugs were used and the dosage (s) administered

Aspirin

Clopidogrel

LMWH

UFH

12. Outcome

28. Did the patient develop thrombotic complications?

No   Yes   Unknown

If yes, please describe the nature of the thrombotic complications (e.g. PTE) and how a diagnosis was reached (e.g. CT or PM)

29. Survival to discharge:
Please select one of the following outcome options

Discharged alive   Died before discharge   Euthanased before discharge

If the patient was euthanased, why was this performed?

30. Survival to 30 days post admission:
Please select one of the following outcome options

Alive at 30 days post admission   Died before 30 days post admission   Euthanased before 30 days post admission

If the patient was euthanased, why was this performed?

31. If the patient died or was euthanased, was a postmortem examination performed?

Yes   No

32. Please use this box to record any other data which you consider pertinent or peculiar about this case.
